# Returning to Sport: Female Athletes Living with and beyond Cancer

**DOI:** 10.3390/ijerph18158151

**Published:** 2021-08-01

**Authors:** Anna L. Schwartz, Christopher M. Terry

**Affiliations:** 1 School of Nursing, Northern Arizona University, 202 E Pine Knoll Dr., Flagstaff, AZ 86011, USA; 2 Sidney Kimmel Cancer Center, Thomas Jefferson University, 834 Chestnut St, Suite 320, Philadelphia, PA 19107, USA; christopher.m.terry15@gmail.com

**Keywords:** cancer, exercise, athletes, competition, long-term effects, late effects, living with and beyond cancer, cancer survivor

## Abstract

Many athletes living with and beyond cancer can continue to train and, in some cases, compete during treatment. Following cancer treatment, athletes can return to competitive sport but need to learn to adapt their physical strength and training to the lingering effects of cancer. It is critical for oncology healthcare providers to use the principles of assess, refer and advise to exercise oncology programs that are appropriate for the individual. Managing side effects of treatment is key to being able to train during and immediately following cancer treatment. Keen attention to fatigue is important at any point in the cancer spectrum to avoid overtraining and optimize the effects of training. Resources are introduced for providers to reference and direct patients to information for psychosocial support and instruction. The purpose of this paper is to present exercise considerations during and after cancer treatment for athletic cancer survivors.

## 1. Introduction

While the incidence of cancer is lower among athletes, athletes get cancer too [1,2]. Many continue to train at some level throughout their treatment. Others take a season or more off to get through treatment, recover and return to a competitive level of fitness and strength. The field of exercise oncology, the study of how physical activity can affect cancer prevention, management and prognosis has grown tremendously over the past decade. Despite the exponential growth in the field, we were only able to identify two studies that focused on athletes living with or beyond cancer. One study was a cross-sectional, descriptive survey of 219 of athletic cancer survivors who continued to exercise throughout their treatment and resumed their sport and competition at the conclusion of treatment [3]. The other study assessed perceptions of treatment-related physical fitness, appearance and identity changes of 22 athletes (18–40 yo) who were receiving chemotherapy and participating in a supervised group exercise intervention comprised of high- and low-intensity physical exercises that included a combination of resistance training, aerobic activities and relaxation sessions [4].

There is a large and convincing body of research demonstrating that exercise is beneficial before, during and after cancer treatment [5,6,7]. As exercise oncology rehabilitation is slowly becoming standard of care, all cancer patients in treatment should be assessed, advised and referred to an exercise program that is appropriate for them: supervised by physiatrist, physical or occupational therapist; community based with an exercise oncology trainer; or home based when a patient is capable of exercising without supervision [6,8,9]. An exercise oncology referral should be part of the treatment plan for everyone living with and beyond cancer and exercise oncology prescriptions need to be tailored to each individual [10]. When working with athletes, the exercise prescription needs to be focused on the specific sport(s) the athlete specializes in, along with any limitations from surgery or the cancer treatment (e.g., colostomy, risk for lymphedema). Perhaps the most important consideration for the fitness professional working with an athlete during the immediate post treatment period is to understand the extent of the physical and emotional side effects that cancer has had on the individual and how that affects their ability to exercise and perhaps their need for specialized exercise with a rehabilitation professional.

Cancer survivors face many barriers to exercise. Studies note that most patients do not receive advice to exercise [11,12] and that clinicians are unaware of value of exercise, lack of awareness of programs to which they can refer patients, and need more education and skills to make referrals to exercise programs [13,14,15,16,17]. While barriers may prevent many cancer survivors from exercising, athletes are different. Exercise is part of their everyday lives and unlike many other cancer survivors, they must learn how to adapt and attenuate their physical activity to their treatment schedule and recovery.

The American College of Sports Medicine (ACSM) Exercise Guidelines for Cancer Survivors is a recommendation for the minimum amount of exercise to prevent cancer, reduce side effects of treatment and improve survivorship [5]. The recommendation is for at least 30 min of aerobic exercise 3 days per week and 2 days a week of resistance exercise. Unless an athlete is extremely compromised by their disease, this amount of exercise will be insufficient to minimize their loss of physical condition. What is key for healthcare practitioners is to assess, advise and refer to appropriate rehabilitation to optimize the athlete’s opportunities to return to competition in the best condition possible. This paper will discuss exercise considerations during and after treatment, in addition to specific training and competition considerations.

## 2. Considerations during Cancer Treatment

Symptom management is key to successfully incorporate exercise training during treatment. Many side effects of treatment (e.g., nausea, vomiting) can be well controlled with medications. All patients need to learn to aggressively treat their symptoms pre-emptively or immediately after onset to minimize their severity. Healthcare teams should provide adequate instruction for optimal utilization of prescribed medications and symptom management.

While keeping a patient active is critical, exercise may need to be placed on hold or modified until a surgical wound is healed. Similarly, exercise may need to be altered if an ostomy is required for a period of time. While waiting for the body to heal, muscle weakness and fatigue may become problematic side effects. Working with a trained exercise oncology rehabilitation team is important to retain as much strength and cardiopulmonary fitness as possible and work around physical limitations and treatment side effects.

Fatigue is the number one problem experienced by people living with and beyond cancer. It is typically most severe during treatment [18]. A survey of 219 athletic cancer survivors found that they exercised an average of 9 h per week, but 69% reported fatigue during active treatment and a concomitant decrease in training level during treatment. However, the majority of respondents reported that exercise attenuated their fatigue [3]. There is now a large and convincing body of research that demonstrates that exercise reduces fatigue both during and following cancer treatment [5,7].

Sun sensitivity is a commonly overlooked side effect that the oncology team needs to discuss as a risk for their athletic cancer patients. Many targeted and immunologic oncology agents (e.g., erlotinib, vemurabinib) can cause significant skin reactions and photosensitivity. Photosensitvity reactions range between 22% to 66% and occur more frequently in the summer [19]. Using broad-spectrum sunscreens with high UVA and wearing sun protective clothing (sunglasses, hat and long sleeves) is important [20]. Alternatively, avoiding the sun and exercising inside whenever possible is probably the safest option to reduce the risk of treatment discontinuation. Even if the sunburn and rashes that are potentiated by the sun do not limit treatment, they can be severe and limit exercise until they are resolved.

Weight change is a disheartening side effect of many cancer regimens. Weight gain increases the risk for recurrence and development of secondary cancers when it is sustained after treatment ends [21,22]. Weight gain is most common in early-stage, non-metastatic, diseases and may impair the performance of an athlete when she is able to return to competition. Discussions about changes in body composition (increase fat, decrease lean body mass and bone density) should be disclosed before treatments begin. Early referral to a nutritionist may help to minimize some of these changes (e.g., increased fat, decreased lean body mass and bone density) along with a well-designed strength training program and psychological support [23].

### Considerations for Specific Acute, Immediate Post-Treatment, Long-Term and Late Effects of Cancer

Cancer and its treatment cause many acute, long-term and late effects that can impair exercise tolerance. Acute side effects occur during treatment. Immediate post-treatment effects begin when treatment ends and may persist for up to 1-year after the completion of treatment. Unique health concerns may linger for many years after treatment (long-term effects) and new side effects of treatment may develop many years after treatment (late effects). Side effects such as nausea, vomiting and fatigue can be acute and occur during treatment or, in the case of cancer-related fatigue, may linger for many years after treatment and be considered a long-term effect [18]. Late effects include, but are not limited to, hypothyroidism, cardiotoxicities and immune deficiencies. The exercise professional working with a survivor during the immediate post-treatment period needs to understand the potential physical and emotional effects of cancer may affect an athlete’s ability to resume exercise training. This is especially true if a treatment regimen was so rigorous that the athlete could not maintain their strength or cardiovascular fitness or is significantly debilitated. Lingering side effects impact each person differently and this must be taken into consideration when developing an exercise prescription.

The following are some of the most common acute, long-term and late effects of treatment:

- Fatigue is the most common side effect both during and following cancer treatment with as many as 90% of patients experiencing fatigue during treatment and 60% of patients having persistent fatigue years after treatment ends [18]. Fatigue that lingers long after treatment ends may be associated with different factors, such as, inactivity, hypothyroidism, cardiac impairment, or depression. Meeting the minimal exercise guidelines as recommended by the ACSM reduces fatigue [5]. Fatigue is further reduced when training is for longer than 30 min per session [5,24,25,26]. However, it is unclear what the optimal or maximum duration of training an athlete with cancer should engage in, and this will be sport-specific. Guidelines may be recommended using parameters of maximal oxygen uptake (VO_2Max_) or maximal heart rate (HR_Max_), both of which can be monitored on a modern smart-device [27]^.^ Fatigue is also commonly measured by validated questionnaires that rely primarily on subjective data from the patient [28]. Fortunately, athletes often have an above-average awareness of their body and its limitations. Fatigue level is a key indicator in determining how hard or intensely one can train and the level of fatigue should be used to modify the training plan.
- Muscle weakness is common and is usually associated with inactivity. Compared to controls, breast cancer survivors who completed chemotherapy had 20–30% less muscle strength [29]. A well-balanced resistance training program can quickly improve and restore the athletes’ muscle strength.
- Lymphedema is common after surgery that involves removal of lymph nodes in the axilla or groin. Disruption of the lymphatic system after surgery can cause a build-up of lymph fluid in the affected area and make movement and use of the affected limb difficult and painful. Upper limb lymphedema occurs in 10% to 90% of breast cancer survivors treated with surgery and/or radiation therapy [30]. Resistance exercise needs to be started in a slow methodical manner to systematically progress from very light weights to heavier weights. Resistance exercise can reduce the number and intensity of flares [31]. Many physical therapists have received additional training certifications to deliver an exercise prescription specifically to address lymphedema.
- Neurotoxicity is associated with chemotherapy agents (e.g., cisplatin, taxanes). These drugs can cause vestibular changes (e.g., vertigo, unsteadiness), ototoxicity, and chemotherapy induced peripheral neuropathy (CIPN). CIPN effects 20–95% of cancer patients and causes numbness and tingling in the hands and/or feet which can be painful and limit functional ability and fine motor skills. CIPN changes can pose significant problems with exercise training and performing fine-motor function, such as descending stairs buttoning a shirt or picking up small items [32]. CIPN may be particularly disruptive to athletes who hold exercise equipment and create a challenge to athletes who play racquet sports, volleyball, throwing or kicking sports. Regimens focused on balance training, endurance and strength training have been shown to reduce symptoms compared to non-trained groups [33]. Balance training appears to have to greatest effect on reduction in CIPN [34].
- Cardiac and pulmonary dysfunction (declines in left ventricular function, congestive heart failure, arrhythmias, arterial stenosis, valvular disease, pulmonary fibrosis, bronchiectasis) are reported after treatment with anthracycline (e.g., doxorubicin), taxanes (e.g., paclitaxel) or bleomycin chemotherapy and mediastinal radiation [35,36]. There is a 5-fold increase in cardiotoxicity among patients treated with anthracycline-based chemotherapy for breast or ovarian cancer, lymphoma, myeloma or sarcoma [37]. While all patients should receive a personalized exercised program, the delivery of such a program may differ depending on the individual. An athlete with minimal exposure to cardio-toxic treatments may obtain clearance for a community-based program, whereas an athlete with greater exposure and a desire to reach higher levels of activity may benefit from clinically supervised exercise. A case study of a multisport female athlete diagnosed with breast cancer describes the effect of exercise training in reversing the declines in cardiorespiratory fitness that occurred during treatment [38]. This required a multidisciplinary discussion, that included the athlete, because cardiopulmonary dysfunction posed a significant threat to her ability to resume competition at a high level. Ultimately, if the healthcare team determines the associated severity or irreversible cardio-toxic side effects should prevent an athlete from safely returning to a specific type or level of activity, the athlete may be encouraged to pursue an alternative type of exercise under the supervision of the care team.
- Endocrine changes can be disrupted by many different cancer treatments. Chemotherapy and radiation therapy can lead to hypothyroidism, infertility, diabetes mellitus, premature menopause and changes in body composition [39,40,41]. Anti-estrogen drugs (e.g., anastrozole, fluvestrant) can cause arthralgias and bone loss. Immunotherapy drugs (e.g., sunitinib) and checkpoint inhibitors (e.g., ipilimumab) can cause profound endocrine dysregulation including adrenal insufficiency, diabetes, thyroid and pituitary dysfunction. Fatigue can be a manifestation of endocrine dysfunction. Exercise may mitigate some of the arthralgias, declines in bone density and negative changes in body composition.
- Early-onset osteopenia and osteoporosis are common and significant consequences of many treatments including steroids (e.g., dexamethasone), chemotherapy (e.g., doxorubicin) and anti-estrogen (e.g., anastrozole, fluvestrant) drugs. These drugs can waste bone and promote the early onset of osteopenia and osteoporosis. While weight-bearing exercise is important for maintaining bone density, if a woman has clinically significant decreased bone mass, interventions that have been most effective have followed the ACSM recommendations to preserve bone health in the general population [41,42]. These exercises should produce moderate to high bone loading forces and include aerobic exercise 3 to 5 days per week and 2 to 3 days a week of resistance exercise. Exercises that cause hyperflexion or hyperextension of the trunk and dynamic twisting are contraindicated in women with osteoporosis [40].
- Impaired immune function may present as a late effect of treatment after bone marrow transplant and B-cell lymphoma. Radiation and immunotherapy drugs (e.g., rituximab) are associated with immunodeficiency disorders [43,44]. Bone marrow transplant and maintenance therapy can lead to late effect impaired immune functions. A literature review of seven studies examining exercise and immune environment in hematologic malignancies suggests exercise favorably influences the immune system by enhancing cellular functions to decrease inflammation but does not prevent impaired immune function [45].

While these long-term and late effects of cancer may impact an athlete, most athletes are able to train and compete without limitations. A multidisciplinary team approach to managing side effects and maximizing the athlete’s ability to regain muscle strength and cardiorespiratory fitness. For example, a woman with breast cancer may have limitations due to lymphedema but by working with a physical therapist and her trainer she should be able to regain much of her upper body strength without causing lymphedema flares. Another example is an athlete who develops CPIN and has difficulty holding heavy weights. This individual would benefit from being directed to use stationary strength equipment to reduce the risk of dropping free weights. There are many simple adaptations that can be made collaboratively with a multidisciplinary team to get an athlete back in their game.

## 3. Training Considerations

When developing a training plan for an athlete, the basic principles of exercise training should be considered to develop the optimal program for complete recovery and success. Periodicity, intensity and specificity should be built into each training cycle. Awareness of changes in physical function due to deconditioning or cancer must be considered when developing training programs. The athlete with cancer may need more days of active rest than their healthy counterparts without cancer. In this population of athletes, it is paramount to maintain a keen awareness of signs of overtraining to guide the athlete and/or coach in modifying training expectations.

It is critical that return to sport considers the safety, functional capacity and functional and physical requirements of the athlete’s sport [46]. Anatomical and functional health status must be considered with strength programs devised to focus on muscle strength to areas of weakness. Rehabilitation professionals should be involved in the care of athletes at this stage to ensure that muscular balance is achieved and full range of motion is attained. The goal of rehabilitation should be to restore normal function as much as possible if there is surgical compromise (e.g., lymphedema, large surgical excision). The athlete may be able to continue training with these physical limitations, but should not be involved in team/contact sports until released from rehabilitation and given instruction that it is safe to return to sport. Individual athletes can return to sport as it is deemed safe by their rehabilitation team.

A well-developed individualized training plan will incorporate active rest into the program to accommodate treatment cycles and the physical and emotional stress that comes with building back to a competitive level. The exercise dose may need to be modified for women with significant debilitation, i.e., start low and go slow. Exercise prescription adaptations should be based on side effects and symptoms not only related to cancer treatment but with attention to the interplay of other co-morbidities. Gradual progression of aerobic and resistance exercise is key to success in optimizing recovery of physical functioning to a level that enables the athlete to fully engage in sport and competition.

## 4. Competition Considerations

Issues related to returning to competition must consider the individual athlete’s physical and emotional condition, along with effects of cancer treatment and the disease itself. While some athletes may return to competition at the level they were at before cancer, others may not be at the same level. These athletes may need support in determining reasonable goals for competition. Readiness for competition is a decision that should be made by the athlete, coach and care team. Realistic expectations should be set for competition so that success is reached and the athlete is not defeated by not attaining previous goals, which can be psychologically destructive.

Special considerations need to be made regarding safety if an athlete wants to compete during cancer treatment. In consultation with their coach and cancer care team, the athlete should discuss their goals. From an oncology perspective, a complete blood count (CBC) and a metabolic panel should be reviewed and monitored to continuously ascertain the patient’s infection risk, bleeding risk, oxygen carrying capacity and electrolyte status. Depending on the level of athlete, data to accurately determine muscle strength and cardiopulmonary condition may help mitigate risk of injury or psychological damage.

Competition after cancer treatment is complete poses fewer risks from an oncology perspective. The athlete, coach and care team should review the current physical and emotional state, fitness, strength and mental readiness for competition. This includes a baseline reassessment, specifically for pertinent long-term or late effects described earlier such as fatigue, muscle weakness, lymphedema, neurotoxicity and cardiopulmonary dysfunction. Typically, there are far fewer reasons to hold an athlete back from competition once they have completed treatment.

## 5. Training and Competition in the Metastatic Setting

Athletes with metastatic disease may feel well enough and desire to continue to remain physically active and engaged in competition to whatever degree they are physically capable of. There is no research on athletic women with metastatic disease. Hence, we can offer no specific recommendation regarding training or competition. However, athletic women with metastatic disease should be encouraged to continue to exercise in a way that improves health, fitness and reduces risks for injury. It should be the athlete’s decision to pursue activities that are meaningful to them and if that means competing in their sport that is the woman’s decision given that she has full understanding of any inherent risks associated with competition and her disease state.

## 6. Recommendations for Practice and Research

Working with athletes with cancer who are returning to sport is both an art and a science. From a clinical perspective, as an athlete, trainer or clinician, astute attention to fatigue and heart rate is necessary and prompt modifications to a training plan are required when fatigue levels increase and/or resting heart rate increases. These subtle changes, when acted on early and the athlete is advised to rest or reduce the training load, may prevent overtraining and promote ongoing improvements in strength and aerobic capacity. While there is a lack of research to guide specific exercise prescriptions or recommendations for athletes returning to sport using principles of exercise training and the exercise oncology exercise guidelines, we believe it is possible to develop sound exercise training programs for athletes returning to sport. Research is needed to recognize patterns of fatigue or heart rate changes that are sport specific to be able to develop a formula or approach to modify a training program.

While the body of research in exercise oncology is growing there is a lack of research on the specifics of exercise prescription for athletes that is specific to different types of sports. Every sport has specific training requirements to return to competitive condition and there is a gap in the knowledge about recommendations for specific preparation for competition and limitations. It could be presumed that this will be individual for each athlete but additional research is needed in this area. The rate at which an athlete can return to competition may be related to tolerance for exercise training during treatment which depends to a great extent on the rigor of the treatment regimen.

## 7. Resources

*Cancer Exercise* is an app developed to help athletes exercise during and following cancer. It is tailored to the athlete’s current state (on/off treatment, type of treatment and daily fatigue level, etc.). *Cancer Exercise* was developed following the principles of exercise physiology, the American College of Sports Medicine exercise guidelines for cancer survivors [5], the extensive body of research in exercise oncology and clinical experience. Dynamic algorithms in the *Cancer Exercise* app create an exercise program that is individualized according to the daily condition of the user. The app is free and is available at: Apps.apple.com/us/app/cancer-exercise/id1520696105 (accessed on 30 July 2021).

*Moving Through Cancer* is an initiative of the American College of Sports Medicine. It provides resources for fitness and healthcare professionals and people living with and beyond cancer. There are easy to use clinical forms to provide an exercise prescription or referral to an exercise oncology program, patient handouts about the benefits of exercise, a list of exercise oncology certified trainers, and a registry of exercise oncology programs around the world. The *Moving Through Cancer initiative* is developing a compendium of exercise oncology programs offered around the world with current contact information. The website, www.exerciseismedicine.org/support_page.php/moving-through-cancer/ is regularly updated.

*Athletes Fighting Cancer* (AFC) uses the power of sport to fight cancer. AFC uses peer navigation in an online forum so athletes can encourage and advise each other in real-time. AFC uses the core values of sport—such as teamwork, mental toughness and understanding your body—to improve lives affected by cancer. AFC believes that anyone who moves their mind and body is an athlete. The organization provides an education center that shares evidence-based information and resources to safely integrate sport into cancer management and survivorship. The AFC website is www.athletesfightingcancer.org (accessed on 30 July 2021).

*MyVictory* is an at-home exercise and community platform for cancer patients, survivors, and caregivers. MyVictory offers live-streamed and on-demand classes and helps users achieve their goals, reduce risk of recurrence and live healthier, more active lives through exercise. The program can be accessed at: www.myvictory.com (accessed on 30 July 2021).

The aforementioned resources are online and available for use throughout the world. At this time, the resources are only available in English. However, they are all being refined and updated to meet the needs of survivors as funding becomes available.

## 8. Conclusions

Continuing or returning to sport and competition during or following cancer treatment is possible for any athlete and many athletes successfully compete during treatment. Key to success in training and competition is mastering symptom management and developing an understanding of the athlete’s body to predict how the athlete will feel after treatment. Even when treatment ends, training may need to be attenuated according to the impact of long-term and late effects of treatment. Special attention to the mental and emotional health of the athlete is essential to optimize performance. Success in the balancing act of training load and symptom management to prevent overtraining is challenging, but maintaining ongoing communication and a healthy relationship between the athlete, coach and care team will provide the foundation for the athlete to excel.

## Data Availability

Not applicable.
